# Sustainable, Fluorine-Free, Low Cost and Easily Processable Materials for Hydrophobic Coatings on Flexible Plastic Substrates

**DOI:** 10.3390/ma12142234

**Published:** 2019-07-11

**Authors:** Carmela T. Prontera, Giuliano Sico, Maria Montanino, Anna De Girolamo Del Mauro, Paolo Tassini, Maria G. Maglione, Carla Minarini, Paola Manini

**Affiliations:** 1 Department of Chemical Sciences, University of Naples Federico II, via Cintia 4, I-80126 Napoli, Italy; 2 Laboratory for Nanomaterials and Devices (SSPT-PROMAS-NANO), ENEA—C. R. Portici, Piazzale Enrico Fermi 1, I-80055 Portici (Napoli), Italy; 3 IMAST S.c.a.r.l., Piazza Bovio, 22, I-80133 Napoli, Italy

**Keywords:** zinc oxide nanoparticles, hydrophobic coating, water vapor transmission rate, green processing

## Abstract

Zinc oxide nanoparticles (ZnONPs) and stearic acid are herein used for the preparation of hydrophobic coatings with good moisture barrier property on flexible plastic substrates. Fast, high throughput, mild and easy-to-run processing techniques, like airbrushing and gravure printing, are applied for thin films deposition of these materials. The results of this study indicated that the best hydrophobic coating in terms of water contact angle (115°) is obtained through a two-steps printing deposition of a ZnONPs layer followed by a stearic acid layer. All the deposition procedures proved to be effective in terms of water vapor barrier properties, reaching values of 0.89 g/m^2^/day, with a 45% reduction with respect to the bare substrate. These preliminary data are very encouraging in the perspective of a low cost and green approach for the realization of functional coatings for packaging applications.

## 1. Introduction

Hydrophobic surfaces have attracted much interest in the last decades for their applications in daily life as well as in some industrial processes. These include, but are not limited to, microfluidic devices [1], self-cleaning [2], anti-corrosion [3], anti-friction [4], buildings exteriors [5], fabrics [6], antimicrobial [7], etc.

The hydrophobicity of a surface is strongly influenced by its composition [8] and by texturing and roughness [9,10]. In this sense, many strategies have been developed to increase the water contact angle (WCA) of the surfaces, such as the deposition of layers of various materials (i.e., fluorine or hydrocarbon compounds [11,12], some types of wax [13] or organic and inorganic materials showing low surface energy [14,15]). In the same regard, a surface showing a hierarchical structure, i.e., the superposition of a nanostructured texturing on a microstructure [8,16], succeeded in getting high hydrophobicity (Figure S3).

Recently, prompted by the search for more sustainable processes and products (most of hydrophobic surfaces are obtained by treatments with fluorine compounds which cause harmful effects to the environment due to their bioaccumulation [17]), particular attention has been paid to the design of bionic surfaces inspired to the natural model of the super-hydrophobicity: the lotus leaf [18]. However, most of the methods reported until now involve high costs or complicated processes and harsh conditions, also preventing the treatment of flexible substrates. Thus, it is still a challenge to develop simple, fast, low-cost, environment-friendly routes for hydrophobic surfaces with controllable morphology to broaden their industrial applications [12,19,20].

To overcome this gap and fulfill the green requisites, herein we report on the preparation of hydrophobic coatings based on the deposition of bio-compatible zinc oxide nanoparticles (ZnONPs) functionalized with stearic acid on flexible polyethylene naphthalate (PEN) substrates.

These materials are readily available, low cost and environmental benign. ZnO is largely used as an additive in rubbers, plastics, ceramics, glasses and pigments, and is one of the most relevant semiconducting material due to its optical and electrical properties [21]. Many studies are reported in the literature concerning the use of ZnO in the preparation of superhydrophobic rigid surfaces, some of which involved the chemical treatment with stearic acid (Figures S1 and S2) [22,23,24,25,26,27]. Lee et al. reported on a facile approach for the fabrication of a superhydrophobicnanocoatings on different substrates (both rigid as silicon and flexible as polyethylene terephthalate (PET)) through a simple but long-lasting procedure based on the spin-coating deposition of a series of layers of ZnONPs, each subjected to a chemical modification step with stearic acid [20]. This study showed how the surface wettability could be modulated by the number of ZnONP coating cycles, by reaching a maximum WCA value of 158° after 15 cycles.

By pursuing a similar approach, herein we report on a simple method to induce a change in the wettability of flexible PEN substrates via a single deposition step. In detail, two different low-cost, high throughput and easy-to-run processing techniques were applied to prepare the coatings, namely, airbrushing (AB) [28] and gravure printing (GP) [29]. Moreover, also the results of two different procedures, one based on the deposition of ZnONPs pre-functionalized with stearic acid (one-step deposition) and another one based on the sequential deposition first of a ZnONPs layer followed by the deposition of a stearic acid layer (two-steps deposition) have been compared.

Finally, the water barrier properties of these hydrophobic coatings have been investigated, as a complementary tool of morphological characterization of the different coatings.

The results confirmed that these materials applied by using such easy process techniques cangreatly improve the hydrophobicity of the PEN substrates and reduce their water vapors transmission rate (WVTR) just after a single deposition of a ZnONPs/stearic acid layer.

## 2. Materials and Methods

### 2.1. Reagents and Substrates

ZnONPs (40 wt.% in ethanol, <130 nm particle size), ZnO nanopowder (<50 nm particle size (BET)), stearic acid and all the solvents were purchased from Sigma Aldrich (St Louis, MO, USA). PEN substrates were DuPont Teijin Films Teonex^®^ Q65FA (Hopewell, VA, USA), thickness 125 µm. The PEN substrates were cleaned according to the following procedure: (1) ultrasound treatment at 70 °C for 60 min after immersion in a solution of Borer Chemie AG Deconex12PA^®^ (Zuchwil, Switzerland) detergent and deionized water (18 MΩ·cm); (2) ultrasound treatment at room temperature for 15 min after immersion in deionized water; (3) immersion in isopropyl alcohol and then in acetone at room temperature; (4) drying in oven in air at 115 °C for more than 2 h before using.

### 2.2. Deposition Technique

The deposition of the ZnONPs/stearic acid coatings has been performed by the gravure printing technique following a previously reported protocol [30] and by the airbrushing technique.

The gravure printer was an IGT G1-5 equipped with an engraved cylinder having a line density of 175 lines/inch, a stylus angle of 120° and a screen angle of 53°, the applied printing force was 700 N and the speed of the samples was 1.0 m/s.

The airbrusher was a manual model Iwata mod. Neo, with gravity-feed dual action airbrush, a 0.35 mm nozzle, compressed air pressure of 3 bar and the distance between the air brusher and the samples was 20 cm; the deposition was performed to fully cover the samples surface.

All the process conditions are listed in Table S1.

### 2.3. ZnO Nanopowder Functionalization with Stearic Acid

A suspension of ZnO nanopowder (100 mg) in toluene (1.5 mL) was treated with stearic acid (20 mg) at 50 °C under vigorous stirring. After 1 h, the reaction mixture was centrifuged (4500 rpm for 15 min) and the sludge was washed with toluene three times to remove the residual stearic acid and finally dried under reduced pressure.

### 2.4. One-Step Deposition Process

A 15 mg/mL solution of stearic acid in ethanol (0.67 mL) was added to a 50 mg/mL suspension of ZnONPs in ethanol (1 mL). The mixture was stirred for 1 h at 50 °C [31] and then diluted with ethanol (4 mL for gravure printing deposition, and 22 mL for airbrushing deposition). To avoid NPs agglomeration and preserve the homogeneity of the deposited films, centrifugation was not performed. These mixtures were then deposited on PEN substrates by using the conditions reported in Table S1. After deposition, all the samples were baked in oven, in air, at 60 °C for 1 h.

### 2.5. Two-Steps Deposition Process

The deposition of the ZnONPs layer on PEN by gravure printing was carried out according to a previously reported procedure [30] by using a 13% *w*/*w* dispersion of ZnONPs in ethanol. For the deposition by airbrushing, a 5 mg/mL dispersion was used.

After these depositions, each sample was baked in an oven, in air, at 140 °C for 60 min, to induce the full evaporation of the solvent. Then, a second deposition step was performed by using an ethanol solution of stearic acid (30 mg/mL for gravure printing and 20 mg/mL for airbrushing was selected as the most favorable concentrations affording high quality thin films). After these depositions, each sample was baked in oven, in air, at 60 °C for 18 h, and then washed with toluene to remove the excess of stearic acid.

### 2.6. Characterizations

Attenuated total reflectance (ATR) spectra of the ZnO nanopowders in transmission mode were recorded with a Nicolet 5700 spectrometer (crystal type: ZnSe, number of scans: 16, resolution: 2 cm^−1^).

Fourier-transform infrared (FT-IR) spectra on thin films have been performed on CaF_2_ slides by using a Perkin Elmer (Waltam, MA, USA) GX instrument (number of scans: 16, resolution: 2 cm^−1^).

Thickness and roughness of the deposited films were investigated by using an optical profilometer Taylor Hobson TalysurfCCI HD (Leicester, UK). The root mean square height (Sq) values of the surfaces roughness were obtained according to the ISO 25178 standard; the reported values are the average results of several measurements.

UV-Vis analysis was performed by using a Perkin Elmer Lambda 900 spectrophotometer.

The surface morphology was investigated by using a scanning electron microscope (SEM) ZEISS LEO 1530 (Oberkochen, Germany); Inlens images with magnification set at 7.4 KX and 1.1 KX collected with EHT = 5 kV).

The WCA was measured by using a Dataphysics OCA20 contact angle system (Filderstadt, Germany) in sessile drop mode [32], and the reported values of WCA are the average of several measurements.

The electrical calcium test was performed to determine the permeation properties of the deposited films [33]. The following experimental set-up was used: a calcium patch and the silver electrodes were evaporated in vacuum, through shadow masks, directly on the films of ZnONPs/stearic acid prepared on PEN. To measure the barrier property of the PEN alone, some devices were prepared also on bare substrates. To select the permeant flow only through the PEN flexible substrates and the deposited films, the calcium sensors were encapsulated by using glass lids glued on PEN with an UV-curable epoxy resin, having barrier properties much higher than the investigated materials [34]. The calcium test sensors were placed inside a climate chamber VötschIndustrieTechnik/Weiss Technik VC3 4018 (Magenta, Italy), to perform the electrical measurements at 38 °C and 90% relative humidity. A Keithley2400 Source Meter (Cleveland, OH, USA) was used for the 4-wire-measurements of the conductance.

The *WVTR* was calculated by means of the following equation:

(1) $$WVTR=-n*\frac{M\left(H_{2}O\right)}{M\left(Ca\right)}*\delta *\rho *\frac{l}{b}*\frac{d\left(\frac{1}{R}\right)}{dt}$$

in which *n* is the reaction ratio of calcium to permeate, *M*(H_2_O) and *M*(Ca) are the molecular weights of water and calcium, *δ* and *ρ* are calcium density and resistivity, *l* and *b* are length and width of the calcium patch, *R* is the electrical resistance of the device and *t* is the time.

The lag time was given by the intersection of the two straight lines tangential to the constant conductance trend and the decreasing conductance trend of the graph. The reported water vapor transmission rates and the lag times are the means of three separate experiments.

## 3. Results and Discussion

To optimize the reaction conditions for ZnONPs functionalization, preliminary experiments were carried out on ZnO nanopowder by using different amounts of stearic acid. The 20% (*w*/*w*) of stearic acid with respect to the ZnO nanopowder proved to be the most convenient ratio affording the desired functionalization against a low amount of unreacted stearic acid. In detail, after 1 h treatment in toluene at 50 °C, it was possible to recover from the reaction mixture a solid exhibiting quite different water dispersion properties; while the starting ZnO nanopowder was finely suspended in water, the solid obtained after treatment with stearic acid floated onto the water surface suggesting the hydrophobic character.

**Figure 1:**
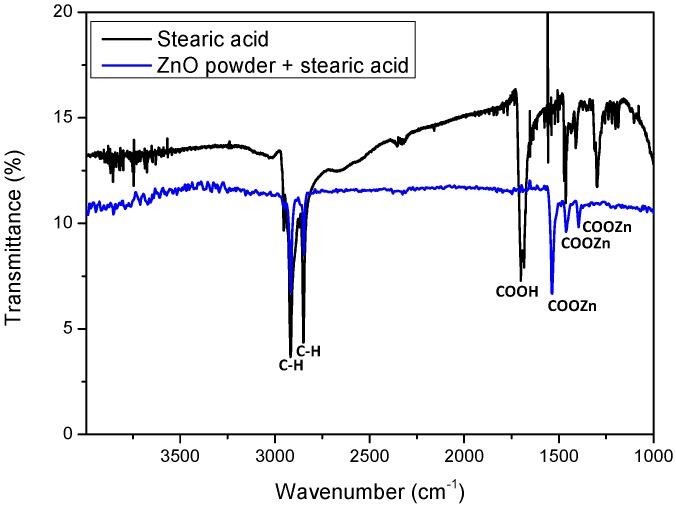
Attenuated total reflectance (ATR) spectra of stearic acid (black trace) and zinc oxide (ZnO) powder after treatment with stearic acid (blue trace).

ATR spectroscopy, carried out on the ZnO nanopowder after the treatment with the stearic acid, confirmed the formation of the zinc stearate functionality and the absence of unreacted stearic acid (Figure 1 and Figure S4). The incorporation of the long alkyl chains on the ZnO nanopowder was supported by the aliphatic C–H stretching vibrations at 2917 and 2849 cm^−1^; moreover, the symmetrical and asymmetrical–COOZn stretching vibrations at 1535, 1462 and 1396 cm^−1^ (–COOH stretching vibration in stearic acid was at 1701 cm^−1^) confirmed that the interaction site of the stearic acid with the ZnO powder was the carboxylic acid functionality [35].

**Figure 2:**
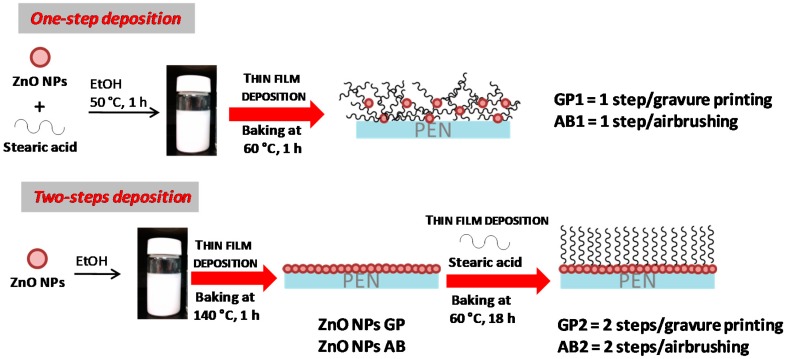
Procedures for the deposition of the zinc oxide nanoparticles (ZnONPs)/stearic acid coatings on polyethylene naphthalate (PEN).

Starting from this evidence, ZnONPs/stearic acid thin films were deposited on PEN substrates according to the procedures (one-step and two-steps processes) schematically reported in (Figure 2).

In brief, in the single step deposition process a suspension of ZnONPs pre-functionalized with the stearic acid was deposited onto the PEN substrates by gravure printing (GP1) or airbrushing (AB1); in the two steps deposition, first a suspension of ZnONPs and then a solution of the stearic acid were deposited separately onto the PEN substrate by gravure printing (GP2) or airbrushing (AB2).

In the two-steps process the functionalization of the layer of ZnONPs with stearic acid occurs only on the outer part of the nanoparticles. On the contrary, in the one-step process, the pre-treatment of ZnONPs with stearic acid makes the nanoparticles fully covered with the alkyl chains, so that the layer deposited on PEN may result more disordered and porous than that obtained with the two-steps process.

FT-IR spectroscopy was performed on the coatings to investigate the chemical nature of the deposited layers and verify the functionalization of the ZnONPs with the stearic acid. The coatings were deposed on infrared transparent CaF_2_ substrates to improve the sensitivity of the measurements. This approach was made possible only for the coatings obtained by airbrushing, because of the high pressure applied during the gravure printing deposition.

**Figure 3:**
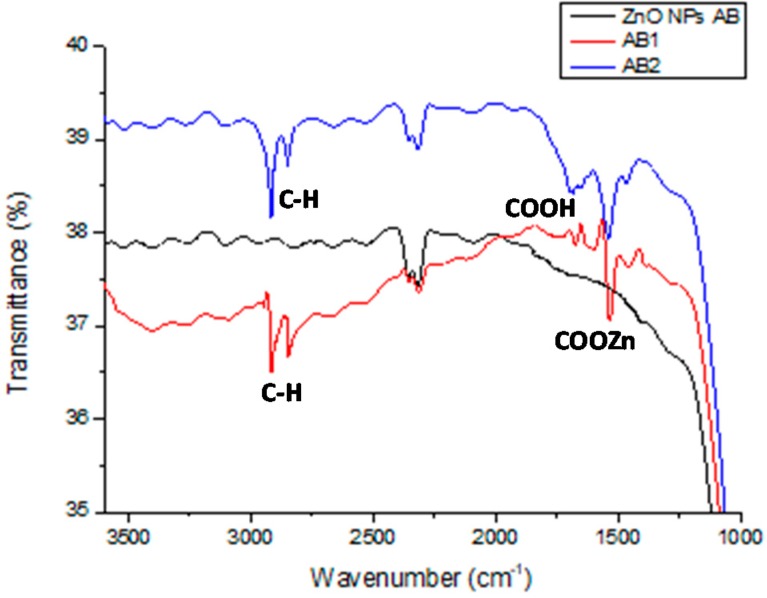
Fourier-transform infrared (FT-IR) spectra on CaF_2_ substrates of the ZnONPs layer deposed by airbrushing (black trace), ZnO NPs/stearic acid AB1 (red trace) and AB2 (blue trace) coatings.

As shown in (Figure 3), the spectra of both AB1 and AB2 coatings exhibited the aliphatic C–H stretching vibrations at 2916 and 2844 cm^−1^ indicative of the presence of the long alkyl chains of the stearic acid, and the stretching vibrations at 1533 and 1461 cm^−1^ of the zinc stearate functionality. A less intense band was visible at 1691 cm^−1^, due to the stretching vibration of the carboxylic group ascribable probably to traces of the stearic acid adsorbed onto the substrate.

All the final coatings (GP1-2 and AB1-2) exhibited a thickness ranging from 140 to 300 nm, with variations in each sample smaller than 30 nm at the various measured points. The roughness proved to be quite low, in the range 10–14 nm, suggesting the potentiality of the chosen processes to obtain good quality films (Table 1). All films were also very transparent in the visible region of the spectrum as shown in (Table 1).

**Figure 4:**
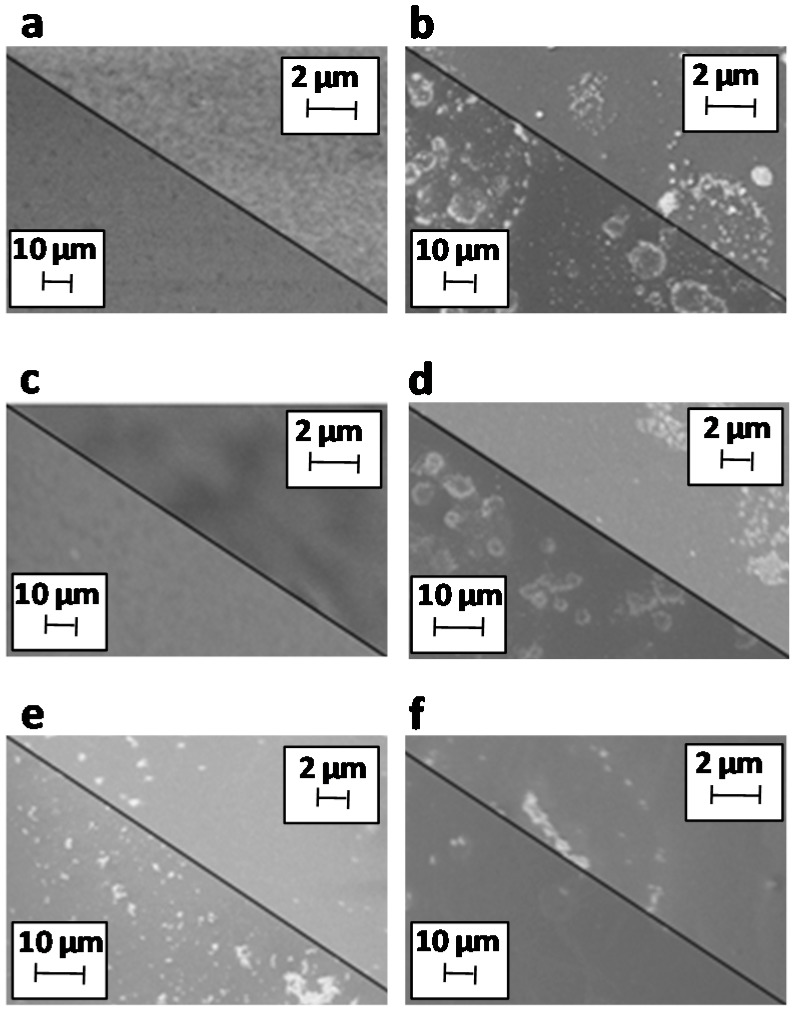
Scanning electron microscope (SEM) images of ZnONPs films obtained by gravure printing (**a**) and airbrushing (**b**), and ZnONPs/stearic acid coatings GP2 (**c**), AB2 (**d**), GP1 (**e**) and AB1 (**f**).

The scanning electron microscopy (SEM) images revealed different morphological features. In the two steps approach, the gravure printing technique deposited smooth films of the ZnONPs, whereas the formation of a large number of visible aggregates was obtained with the airbrushing technique (Figure 4a,b). As reported in the literature, this can be ascribed to the deposition mode; in particular, the evaporation of the solvent of the droplets deposited via the airbrushing technique may induce the formation of ZnONPs aggregates on the substrate [36,37].

The subsequent deposition of the stearic acid layer did not affect the smoothness of the coating obtained by gravure printing (Figure 4c), but slightly reduced the number of aggregates in the airbrushing mode probably by uniformly covering the ZnONPs surface (Figure 4d).

A significant reduction of the aggregates formation was observed with the one-step approach by using the airbrushing technique, probably due to the effect of the stearic acid, that can remarkably improve the dispersion of the ZnONPs in organic solvents and prevent their agglomeration during the droplet evaporation (Figure 4e,f).

The WCA measurements carried out on all the coatings showed interesting results. The single layers of ZnONPs coating, obtained via a single gravure printing or airbrushing deposition, exhibited very different WCA values: 26° and 100°, respectively (Table 2).

These data reflect the morphological differences observed in the SEM images (Figure 4a–b). As reported in the literature, the smooth and packed printed ZnONPs coating conferred to the PEN surface a more hydrophilic character (WCA from 68° to 26°) as a consequence of the presence of –OH residues [38]. The higher WCA value measured for the airbrushed ZnONPs coating is probably due to the presence of the aggregates, leading to the formation of a more porous coating. This situation can be explained in terms of the metastable Cassie–Baxter state, in which the water permeation and adhesion is prevented by the air occupying the porous cavities [39].

The deposition of the layer of stearic acid on the ZnONPs films in the two-steps process led, in the case of the gravure printing technique, to a marked increase in the hydrophobic character of the surface (GP2 WCA from 26° to 115°), whereas no appreciable effect was observed in the case of the airbrushing deposition (AB2 WCA from 100° to 102°).

The one step deposition approach of the ZnONPs/stearic acid layer also proved to be effective in conferring to the PEN substrate a hydrophobic character: in this case, both the deposition techniques led to quite similar WCA values (99° for GP1 and 95° for AB1).

In general, even if the differences proved in some cases not to be significant, there is a certain tendency of the WCA values of the coatings obtained with the two-steps deposition to be lower than the ones obtained with the one-step deposition, as a result of the more regular hydrophobic outer layer (Figure 2).

Overall, these data pointed out the more hydrophobic character obtained with the deposition procedures reported herein and based on gravure printing and airbrushing, with respect to those reported in the literature for the same ZnONPs/stearic acid layers deposed by spin coating [20].

In another set of experiments the effects of the coatings on the WVTR and the lag time in the permeation process of the PEN substrate were determined [40]. These parameters were evaluated by using the electrical calcium test [32,41]. In brief, the water permeant transmission rate through a barrier is estimated thanks to the variation in the electrical resistance induced by the reaction between a metallic calcium patch, sealed using the barrier under test, and the permeated water, while keeping the system in constant environmental conditions of temperature, pressure and humidity (Figure S5). In the presence of water, the conductive and opaque metallic calcium reacts, forming calcium hydroxide, which is typically insulating and transparent. By measuring the electrical resistance of the calcium patch with the time it is possible to calculate the WVTR and the lag time (see Materials and Methods section).

In order to exclude the contribution of the contact resistances to the measurements of each Ca patch resistance, a four-wires sensing geometry was used (Figure S9) [42,43].

The plots of the normalized conductance versus time, for the various samples, are shown in the Supplementary Material section (Figures S6–S8), in which is evident the longer time of the first part of the graph at constant electrical conductance, giving the increase of the lag time.

In Table 3, the WVTR and the lag time for each of the coatings are reported. As it can be seen, the applied coatings produced evident decreases of the WVTR and increases of the lag time respect to the bare PEN. This behavior can be rationalized considering that the permeation process is a multistage phenomenon, in which the permeant molecules pass through a film under a gradient of concentration [40]. In the first stage, the permeant condenses on the film surface (adsorption); in the second stage, the dissolution and diffusion through the barrier layer occur. According to the morphological characterization, the observed VWTR reduction is essentially due to the exclusion of part of the samples area to the water vapor permeation process and slight tortuosity effect on the diffusion path affecting the transient process providing an increase in the lag time.

The most effective results in terms of WVTR reduction of the substrates were obtained with both the deposition approaches. The GP1 and AB1 coatings proved to be more efficient than the GP2 and AB2 ones, leading to up to a 45% reduction of the WVTR (0.89 g/(m^2^·d)) and up to a 100% of increase of the lag time (2.98 h) with respect to the PEN substrate (1.58 g/(m^2^·d) and 1.51 h).This is probably due to the one step deposition process itself, which combines the hydrophobic property of the stearic acid, representing the external layer of the coating, and the structural disorder of the deposited layer (Figure 2), determining the formation of complex permeation paths that increase both the barrier parameters.

Overall, the WVTR values measured for the ZnONPs/stearic acid coatings reported herein are comparable to those measured for other coatings commonly used for packaging applications based on biopolymers (i.e., thermoplastic starch) or fossil oil (i.e., ethylene vinyl alcohol) [44]. Worthy of note is also the fact that, whatever the deposition technique, the moisture barrier properties exhibited by the single couple ZnONPs/stearic acid proved to be comparable to a two-pair SiOx/SiCxHy coating [45] and more efficient than some representative hybrid multilayer coatings based on 2-hydroxy-3-trimethylammoniumpropylchloride starch/montmorillonite and chitosan/montmorillonite (20 bilayer each) [46].

## 4. Conclusions

In this work, it has been demonstrated that it is possible to use a green and low cost approach, both for the materials and the applied process techniques, to obtain hydrophobic and moisture barrier coatings on flexible plastic substrates with potential application on a large scale.

The performances of the ZnONPs/stearic acid coatings have been explored by using two different deposition procedures (one step or two steps) and two easy and low-cost solution based techniques (gravure printing and airbrushing). A general improvement of the hydrophobic character and of the barrier properties was observed for all the tested coatings respect to the bare PEN substrates.

The best results in term of hydrophobicity and barrier properties were obtained by using a one-step approach; moreover, the airbrushing deposition seemed to be the most suitable processing technique, allowing the realization of functional coatings even on substrates with a complex curvature.

Overall, the results reported herein, obtained with a single deposition of a ZnONPs/stearic acid layer, open the perspectives toward the preparation of low cost and sustainable functional coatings for flexible plastic substrates with good hydrophobic and moisture barrier properties, which can be very attractive for packaging applications [45,47].

## Figures and Tables

**Table 1:** Morphological and optical characteristics of the ZnONPs/stearic acid coatings.

	Optical Transmittance (%, @400–800 nm)	Thickness (nm)	Roughness (Sq, nm)
PEN	99–98	-	12.3 ± 2.2
ZnONPs (GP)	99–98	232 ± 10	10.6 ± 1.6
ZnONPs (AB)	99–98	115 ± 15	8.4 ± 1.2
GP2	98–97	305 ± 12	12.1 ± 1.5
AB2	99–98	160 ± 24	10.3 ± 2.2
GP1	99–98	240 ± 11	13.3 ± 1.8
AB1	99–98	140 ± 21	14.2 ± 1.7

**Table 2:** Contact angle characterization of the coatings.

	Water Contact Angle (°)	Contact Angle Image
PEN	68 ± 5	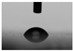
ZnONPs (GP)	26 ± 4	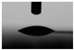
ZnONPs (AB)	100 ± 3	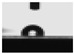
GP2	115 ± 4	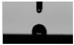
AB2	102 ± 6	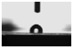
GP1	99 ± 3	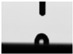
AB1	95 ± 6	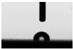

**Table 3:** Water vapor transmission rate (WVTR) and lag time of the coatings, determined through electrical calcium tests carried out in a climatic chamber at 38 °C and 90% of relative humidity.

	WVTR (g/(m^2^·d))	Lag Time (h)
PEN	1.58 ±0.13	1.51 ± 0.32
ZnONPs (GP)	1.18 ± 0.11	2.13 ± 0.45
ZnONPs (AB)	1.24 ± 0.15	2.33 ± 0.41
GP2	1.27 ± 0.18	2.47 ± 0.28
AB2	1.02 ± 0.20	2.26 ± 0.33
GP1	0.89 ± 0.16	2.63 ± 0.38
AB1	0.96 ± 0.21	2.98 ± 0.42

## Supplementary Materials

The following are available online at https://www.mdpi.com/1996-1944/12/14/2234/s1, Figure S1: Formula of stearic acid, Figure S2: Chemical reaction for the functionalization of ZnONPs with stearic acid, Figure S3: Effect of the surface structuring on the hydrophobicity, Figure S4: ATR spectrum of pure ZnO nanopowder, Figure S5: Scheme of the experimental setup for the electrical calcium test, Figure S6: Conductance versus time for the electrical calcium test carried out on ZnONPs coatings on PEN, Figure S7: Conductance versus time graph for the electrical calcium test carried out on ZnONPs/stearic acid coatings on PEN obtained by gravure printing, Figure S8: Conductance versus time graph for the electrical calcium test carried out on ZnONPs/stearic acid coatings on PEN obtained by airbrushing, Figure S9: Four-wires sensing geometry for electrical calcium test measurements, Table S1: Parameters used for the deposition of the ZnONPs/stearic acid coatings.
