# Supplementary material for: Sustainable, Fluorine-Free, Low Cost and Easily Processable Materials for Hydrophobic Coatings on Flexible Plastic Substrates
Source: Materials (Basel). 2019 Jul 11;12(14):2234. doi: 10.3390/ma12142234 (PMC6678896; doi:10.3390/ma12142234)
Supplement: Supplementary file 1 [file materials-12-02234-s001.pdf]

materials

Supplementary Materials: Sustainable, Fluorine-Free, Low Cost and Easily Processable Materials for Hydrophobic Coatings on Flexible Plastic Substrates

Carmela T. Prontera, Giuliano Sico, Maria Montanino, Anna De Girolamo Del Mauro, Paolo Tassini, Maria G. Maglione, Carla Minarini and Paola Manini

Figure S1: Formula of stearic acid. S2

Figure S2: Chemical reaction for the functionalization of ZnO NPs with stearic acid. S2

Figure S3: Effect of the surface structuring on the hydrophobicity S2

Figure S4. ATR spectrum of pure ZnO nanopowder S2

Table S1: Parameters used for the deposition of the ZnO NPs/stearic acid coatings. S3

Figure S5: Scheme of the experimental setup for the electrical calcium test. S4

Figure S6: Conductance *vs* time graph for the electrical calcium test carried out on ZnONPs coatings on PEN. S4

Figure S7: Conductance *vs* time graph for the electrical calcium test carried out on ZnONPs/stearic acid coatings on PEN obtained by gravure printing. S5

Figure S8: Conductance *vs* time graph for the electrical calcium test carried out on ZnONPs/stearic acid coatings on PEN obtained by airbrushing. S5

Figure S9: 4-Wires sensing geometry for electrical calcium test measurements S6

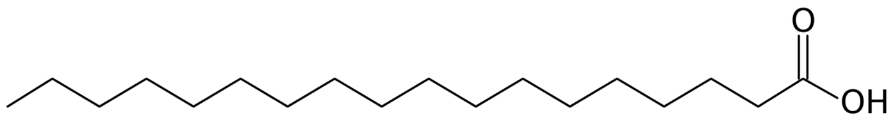

Figure S1. Formula of stearic acid.

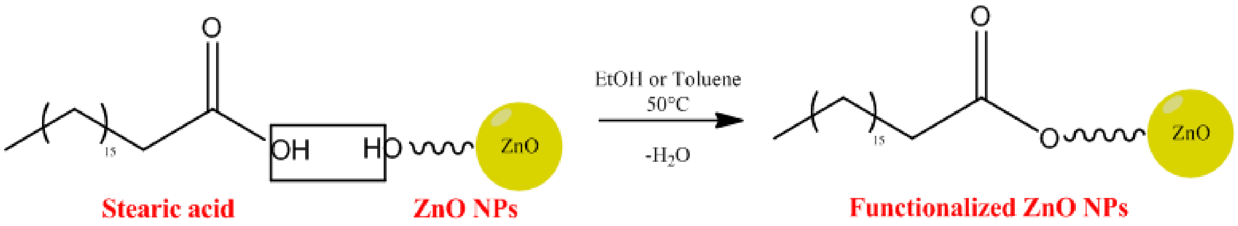

Figure S2. Chemical reaction for the functionalization of ZnONPs with stearic acid.

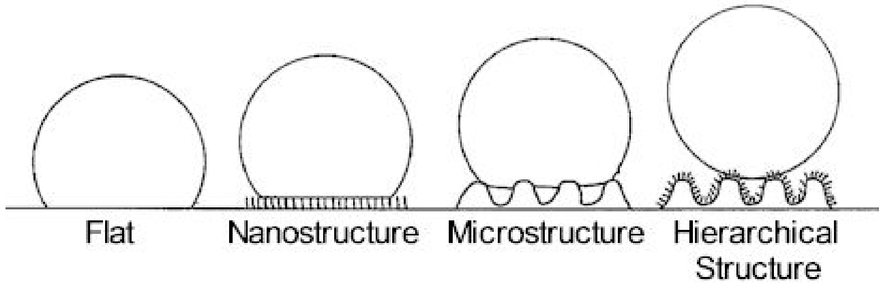

Figure S3. Effect of the surface structuring on the hydrophobicity, which increases from left to right.

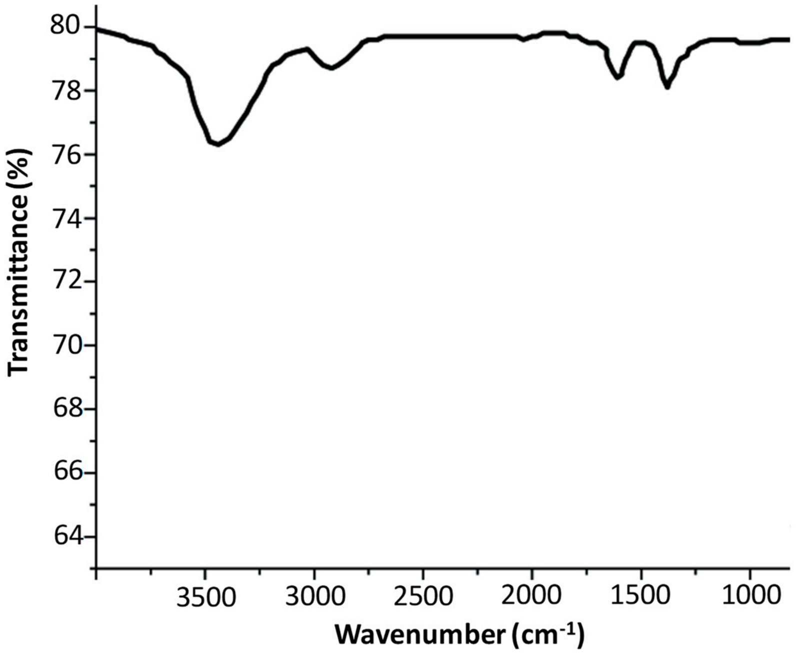

Figure S4. ATR spectrum of pure ZnO nanopowder.

Table S1. Parameters used for the deposition of the ZnONPs/stearic acid coatings.

**Process**	**1st step**	**Thermal treatment**	**2nd step**	**Thermal treatment**
**GP2**	ZnONPs (13% w/w in EtOH)<br><br>**Gravure Printing parameters:**<br>printer IGT G1-5;<br>engraved cylinder line density = 70lines/cm;<br>stylus angle = 120°;<br>screen angle = 53°;<br>printing force = 700 N;<br>printing speed = 60 m/min	140 °C, in oven, in air, for 1 h	Stearic acid (30 mg/mL in EtOH)<br><br>**Gravure Printing parameters:**<br>printer IGT G1-5;<br>engraved cylinder line density = 70 lines/cm;<br>stylus angle = 120°;<br>screen angle = 53°;<br>printing force = 100 N;<br>printing speed = 12 m/min	60 °C, in oven, in air, for 12 h
**AB2**	ZnONPs (5 mg/mL in EtOH)<br><br>**Airbrushing Painting parameters**:<br>Iwata mod. Neo<br>Nozzle size = 0,35 mm<br>Pressure = 3 bar<br>Working distance = 20 cm.	140 °C, in oven, in air, for 1 h	Stearic acid (20 mg/mL in EtOH)<br><br>**Airbrushing Painting parameters:**<br>Iwata mod. Neo<br>Nozzle size = 0,35 mm<br>Pressure = 3 bar<br>Working distance = 20 cm.	60 °C, in oven, in air, for 12 h
**GP1**	ZnO + stearic acid in EtOH (10 mg/mL)<br><br>**Gravure Printing parameters:**<br>printer IGT G1-5;<br>engraved cylinder line density = 70 lines/cm;<br>stylus angle = 120°;<br>screen angle = 53°;<br>printing force = 500 N;<br>printing speed = 60 m/min	60 °C, in oven, in air, for 1 h	-	-
**AB1**	ZnO+ stearic acid in EtOH (2.5 mg/mL)<br><br>**Airbrushing Painting parameters:**<br>Iwata mod. Neo<br>Nozzle size = 0,35 mm<br>Pressure = 3 bar<br>Working distance = 20 cm	60 °C, in oven, in air, for 1 h	-	-

GP1: gravure printing one-step process; AB1: airbrushing one-step process; GP2: gravure printing two-step process; AB2: airbrushing two-step process.

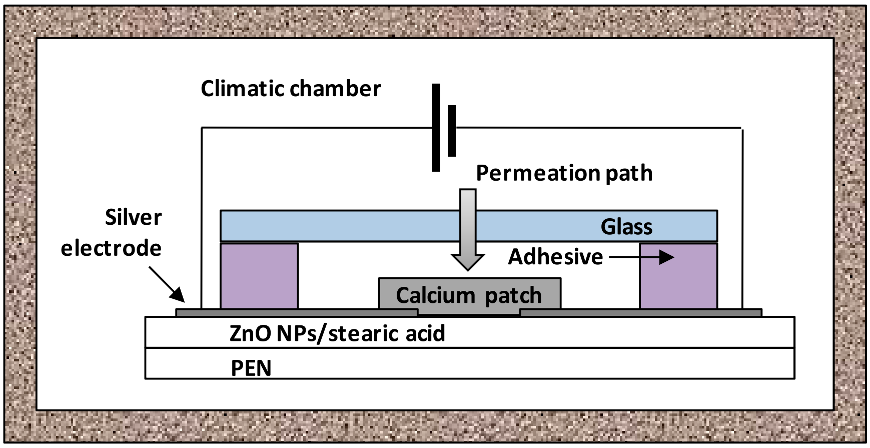

Figure S5. Scheme of the experimental setup for the electrical calcium test. The thick external rectangle represent the climatic chamber.

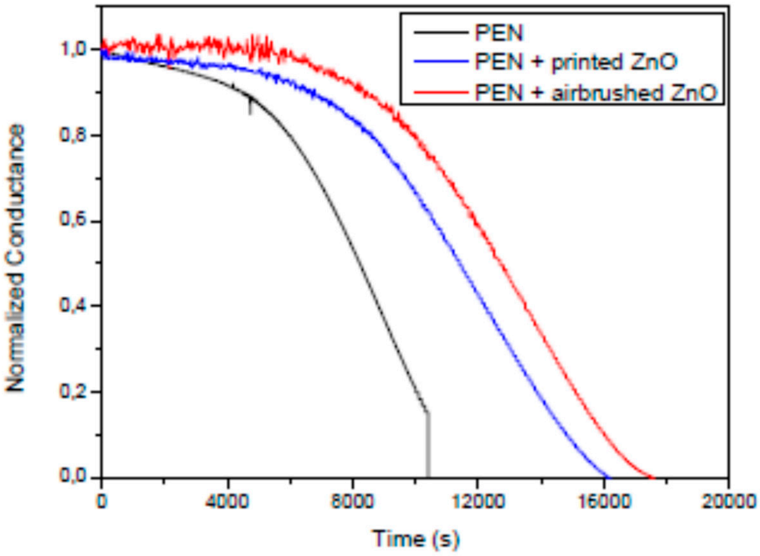

Figure S6. Normalized conductance *vs* time for the electrical calcium test carried out on bare PEN (black trace), and on ZnONPs coatings on PEN obtained by gravure printing (blue trace) and by airbrushing (red trace).

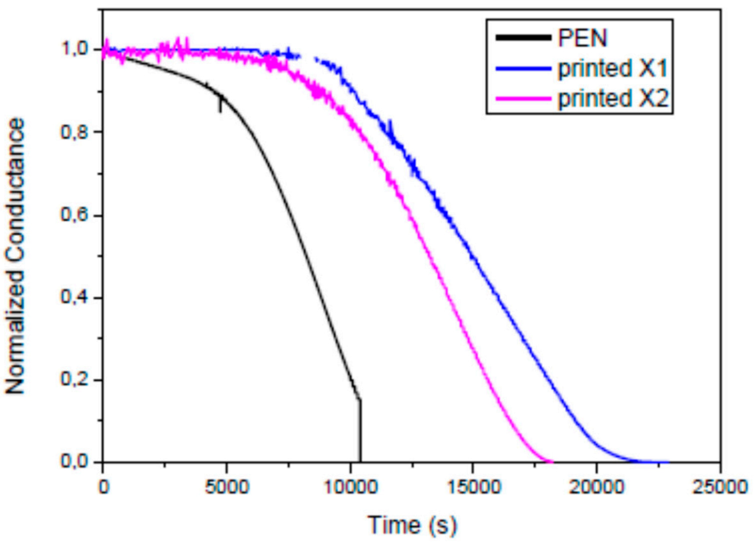

Figure S7. Normalized conductance *vs* time for the electrical calcium test carried out on bare PEN (black trace), and on ZnONPs/stearic acid coatings on PEN obtained by the gravure printing one step (GP1, blue trace) and two steps (GP2, magenta trace) approach.

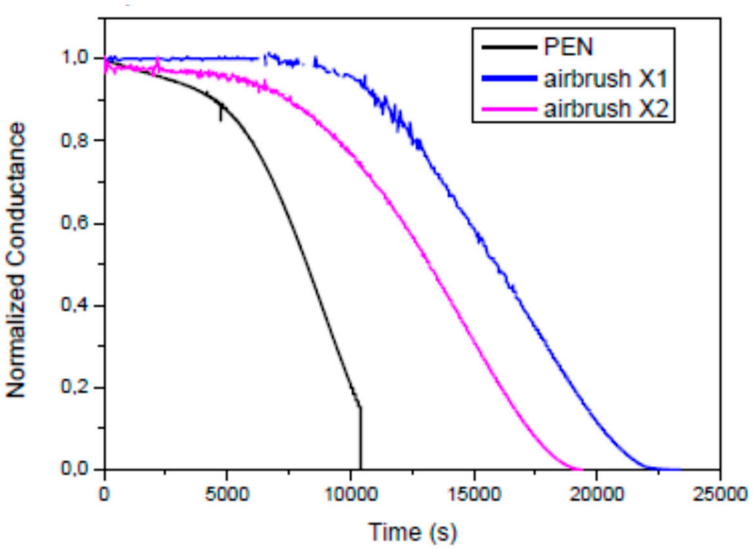

Figure S8. Normalized conductance *vs* time for the electrical calcium test carried out on bare PEN (black trace), and on ZnONPs/stearic acid coatings on PEN obtained by the airbrushing one step (AB1, blue trace) and two steps (AB2, magenta trace) approach.

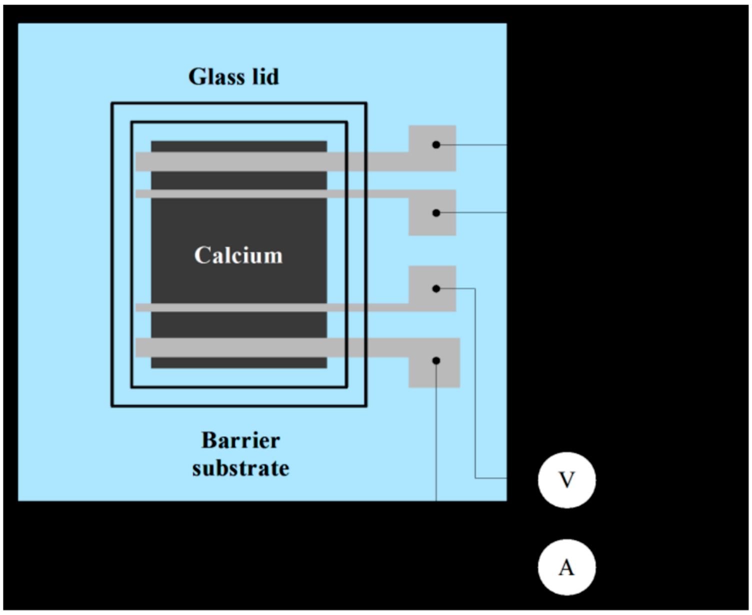

Figure S9. 4-Wires sensing geometry for electrical calcium test measurements.

© 2019 by the authors. Submitted for possible open access publication under the terms and conditions of the Creative Commons Attribution (CC BY) license (http://creativecommons.org/licenses/by/4.0/).
